# Treatment Options for Troublesome Itch

**DOI:** 10.3390/ph15081022

**Published:** 2022-08-19

**Authors:** Sumika Toyama, Mitsutoshi Tominaga, Kenji Takamori

**Affiliations:** 1 Juntendo Itch Research Center (JIRC), Institute for Environmental and Gender-Specific Medicine, Juntendo University Graduate School of Medicine, 2-1-1 Tomioka, Urayasu 279-0021, Chiba, Japan; 2 Anti-Aging Skin Research Laboratory, Juntendo University Graduate School of Medicine, 2-1-1 Tomioka, Urayasu 279-0021, Chiba, Japan; 3 Department of Dermatology, Juntendo University Urayasu Hospital, 2-1-1 Tomioka, Urayasu 279-0021, Chiba, Japan

**Keywords:** acupuncture therapy, intractable pruritus, itch, itch inhibitory mechanism, oral therapy, topical medication, troublesome itch, phototherapy

## Abstract

Itch (or pruritus) is an unpleasant sensation, inducing the desire to scratch. It is also a major and distressing symptom of many skin and systemic diseases. The involvement of histamine, which is a major itch mediator, has been extensively examined. Recent studies suggest that histamine-independent pathways may play roles in chronic itch. Therefore, antihistamines are not always effective in the treatment of patients with chronic itch. The development of biologics and κ-opioid receptor (KOR) agonists has contributed to advances in the treatment of itch; however, since biologics are expensive for patients to purchase, some patients may limit or discontinue their use of these agents. Furthermore, KOR agonists need to be prescribed with caution due to risks of side effects in the central nervous system. Janus kinase (JAK) inhibitors are sometimes associated with side effects, such as infection. In this review, we summarize antidepressants, antineuralgics, cyclosporine A, antibiotics, crotamiton, phosphodiesterase 4 inhibitor, botulinum toxin type A, herbal medicines, phototherapy, and acupuncture therapy as itch treatment options other than antihistamines, biologics, opioids, and JAK inhibitors; we also explain their underlying mechanisms of action.

## 1. Introduction

Itch (or pruritus) is an unpleasant sensation that induces the desire to scratch, which reduces the quality of life (QOL) of patients [1]. Histamine is one of the major itch mediators and has been employed as the main agent in antipruritic therapies. However, antihistamines often lack efficacy in patients with chronic itch [2,3,4]. Intractable pruritus, for which antihistamines are ineffective, is a common symptom of skin diseases, such as atopic dermatitis (AD), psoriasis, and prurigo nodularis. Intractable pruritus also affects some patients with systemic diseases, including renal diseases [5], cholestasis liver disorder [6], and bowel diseases [7]. The lack of a response to high-potency antihistamines of different types in patients with chronic itch suggests the involvement of other mediators.

κ-opioid receptor (KOR) agonists (e.g., nalfurafine) have been shown to suppress pruritus in patients with chronic renal failure, and in patients with cholestasis [8]. However, nalfurafine is associated with the development of side effects in the central nervous system (CNS) [9], and is thus prescribed with caution. Dupilumab, a monoclonal antibody that specifically binds to the shared alpha chain subunit of the interleukin (IL)-4 and IL-13 receptors, was shown to improve clinical end points, including the attenuation of pruritus in AD [10]. However, biological products are expensive for patients to purchase, thus some patients may limit or discontinue their use. JAK inhibitors have also been suggested to ameliorate itching effectively. However, they are also associated with side effects, such as infection [11,12,13]. Although emollients also partly control intraepidermal nerve density, they have more of a preventive than a therapeutic effect. Therefore, it is necessary to control itch from a multifaceted perspective through the development of new treatments and drug repositioning.

In this review, we summarize alternative treatment options for troublesome itch, including antidepressants, antineuralgics, cyclosporine A, antibiotics, crotamiton, phosphodiesterase 4 (PDE_4_) inhibitor, herbal medicines, phototherapy, and acupuncture therapy (Table 1). This review may inform physicians seeking treatment options for patients unresponsive to mainstream itch treatments, such as KOR agonists, biological products, and JAK inhibitors.

## 2. Pharmacotherapeutic Options for Itch

### 2.1. Antidepressants

Antidepressants include tricyclics, tetracyclics, and serotonin noradrenaline reuptake inhibitors (SNRIs). Serotonin (5-hydroxytryptamine, 5-HT), which is produced by mast cells, basophils, and platelets, plays a role in itch in a transient receptor potential vanilloid 4 (TRPV4)-dependent manner via the 5-HT receptor. The antipruritic effects of selective serotonin reuptake inhibitors (SSRIs) have been investigated. SSRIs are safer than tricyclics and are currently the most widely used antidepressants [14,15,16].

Paroxetine (trade names include Aropax (GlaxoSmithKline, London, UK) among others), an SSRI, has been reported to exert antipruritic effects in cancer patients and on itch associated with true erythrocytosis and psychogenic diseases [17,18,19]. Paroxetine and fluvoxamine (Neuraxpharm, Langenfeld, Germany) were also shown to suppress itch in patients with severe chronic pruritus [20]. In pediatric patients with pruritus due to cholestasis, sertraline (Zoloft; Pfizer, NY, USA) attenuated itch and improved sleep quality [21].

A previous study demonstrated that mirtazapine (Remeron; Organon, NJ, USA), a noradrenergic and specific serotonergic antidepressant (NaSSA), significantly attenuated itch in tumor-associated cutaneous pruritus, and in pruritus at night without depression [22].

The intrathecal administration of milnacipran (Sigma, St. Louis, MO, USA), an SNRI, suppressed serotonin-induced itch in mice [23]. Therefore, antidepressants, such as SSRIs, SNRIs, and NaSSAs, may be useful for intractable itch by inhibiting the reuptake of the itch mediator serotonin; however, their antipruritic mechanisms have not yet been elucidated in detail. Serotonin binds to serotonin receptors on sensory nerves, which express TRPV4, inducing itching. However, the endogenous analgesic mechanism is exhibited by activating the descending inhibitory system in the CNS. A similar inhibitory mechanism may also reduce itch [24].

However, antidepressants are off-label and take a long time to exert therapeutic effects, and the risk of side effects (digestive symptoms, such as nausea; psychiatric symptoms, including anxiety; CNS depression; eye symptoms, such as dry eye and inflammation; and skin symptoms) needs to be considered [25,26,27,28].

### 2.2. Antineuralgics

A common feature of skin diseases that present with intractable itch is abnormal skin barrier function. The skin barrier prevents the entry of foreign substances and evaporation of water; however, when it is compromised by environmental or genetic factors, sensory nerve fibers penetrate the epidermis, causing hypersensitivity to itch [29]. Neuropathic itch is considered to involve a similar mechanism to that of neuropathic pain, which involves peripheral sensory nerve hypersensitivity and decreased capacity to suppress calcium ion influx and neurotransmitters, such as substance P (SP) and calcitonin gene-related peptide (CGRP), release to itch-dedicated interneurons in the spinal dorsal horn [30]. Therefore, pregabalin, which is effective against neuropathic pain, is also a useful treatment for neuropathic pruritus [30]. Pregabalin (Lyrica; Pfizer) was previously shown to suppress itch in patients with uremia, cancer, erythrocytosis, prurigo nodularis, and post-burn [31]. It has been suggested to exert its analgesic effects in the CNS by inhibiting calcium influx and N-methyl-D-aspartate (NMDA) receptors through binding to the α2δ subunit, which plays an auxiliary role in the function of voltage-gated calcium channels, and by suppressing the release of neurotransmitters, such as noradrenaline, glutamate, glycine, γ-aminobutyric acid (GABA), SP, CGRP, and acetylcholine [32,33,34]. Therefore, pregabalin is speculated to inhibit neurotransmitter release and exert antipruritic effects in the CNS by inhibiting calcium influx and NMDA receptors via binding to the α2δ subunit.

On the other hand, mirogabalin (Tarlige; Daiichi Sankyo Co., Ltd., Tokyo, Japan) was found to inhibit calcium influx in the peripheral nervous system and suppress neurotransmitter release, resulting in analgesia [35]. Therefore, mirogabalin may be useful for peripheral itch. However, these antineuralgics are off-label for itch and their application needs to be explained to patients.

**Figure 1:**
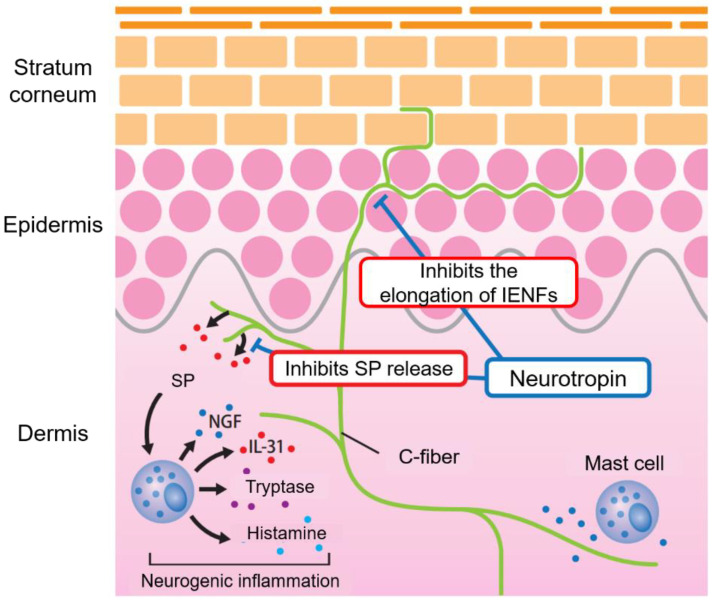
Antipruritic mechanism of NTP for intractable itch in AD. NTP inhibits SP release from nerve endings, which presumably suppresses neurogenic inflammation caused by nerve-SP-mast cells. In addition, NTP inhibits the elongation of IENFs and reduces itch hypersensitivity.

Neurotropin (NTP, Nippon Zoki Pharmaceutical Co., Ltd., Osaka, Japan), a non-protein extract isolated from the inflamed skin of rabbits inoculated with vaccinia virus, is widely used in Japan and China to treat various chronic pain conditions. We previously reported the antipruritic effects of NTP following its intraperitoneal administration to AD model mice and a control group [36]. The antipruritic mechanism was attributed to the suppression of neurogenic inflammation via the inhibition of SP release from nerve endings and reduction in itch hypersensitivity by the prevention of intraepidermal nerve fiber (IENF) elongation [37,38] (Figure 1).

### 2.3. Cyclosporine A

Cyclosporine A (CsA, Neoral; Novartis, Basel, Switzerland), a calcineurin inhibitor, is an immunosuppressant that is widely used in the treatment of inflammatory diseases. Its efficacy against AD symptoms, including itch, has been demonstrated and it has been approved for the treatment of AD [39].

**Figure 2:**
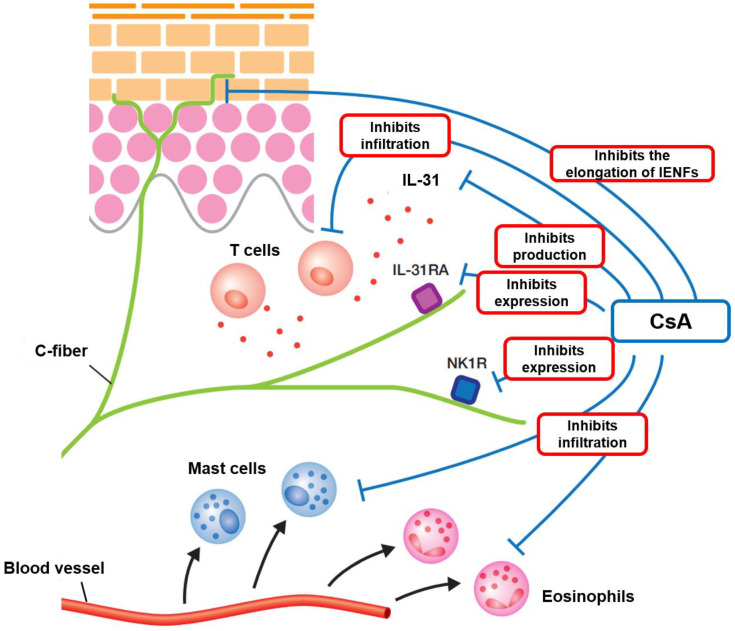
Antipruritic mechanism of CsA for intractable itch in AD. CsA down-regulates expressions of IL-31RA and NK1R in peripheral nerves. CsA also inhibits the infiltration of immune cells, such as CD4^+^ T cells, mast cells, and eosinophils. In addition, CsA reduces the perception of itch stimuli by suppressing the proliferation of IENFs.

Previous studies have shown that IL-31 receptor A (IL-31RA) and NK-1 receptor (NK-1R) mRNA expression levels in dorsal root ganglion (DRG) of AD mice are significantly increased compared to DRG of control mice, and that these increases are significantly suppressed by CsA treatment. In addition, CsA suppressed epidermal thickness and the infiltration of immune cells, such as IL-31^+^ cells, CD4^+^ T cells, mast cells, and eosinophils, as well as the proliferation of IENFs [40] (Figure 2).

Skin lesions and blood IL-31 levels were significantly increased in AD patients and positively correlated with scores on a tool that assesses severity of AD (Severity SCORing Atopic Dermatitis; SCORAD), while CsA decreased blood IL-31 levels, which correlated with reduction in disease severity and the attenuation of itch [41].

CsA effectively reduced disease severity, attenuated itch, and improved QOL in patients with psoriasis [42]. Based on these findings, CsA may effectively ameliorate itch and inflammation not only in AD, but also in psoriasis. However, CsA dosing may be restricted in patients with liver or kidney damage who are taking colchicine, which is contraindicated.

### 2.4. Antibiotics

An abnormal composition of bacterial species, called dysbiosis, was found in AD skin lesions, and *Staphylococcus aureus* (*S. aureus*) was detected [43]. *S. aureus* colonization has been shown to enhance Th2 cell differentiation in AD [44]. Macrolide antimicrobials are known to exhibit not only antibacterial activity but also anti-inflammatory and immunomodulating activities and are presumed to be effective against various skin diseases. A recent study reported that the application of ointment containing josamycin (Adipogen, Liestal, Switzerland) to the skin of AD mice attenuated dermatitis, reduced the number of *S. aureus* by inhibiting *S. aureus* colonization and Th2 cell differentiation, down-regulated IL-31 expression, and suppressed itch in skin [45]. Furthermore, roxithromycin (Rulid, Sanofi, Paris, France) and clarithromycin (Brand name Biaxin, (Abbott Laboratories, IL, USA) among others) ameliorated itch in antihistamine-resistant AD patients and patients with psoriasis and chronic pruritus [46].

**Figure 3:**
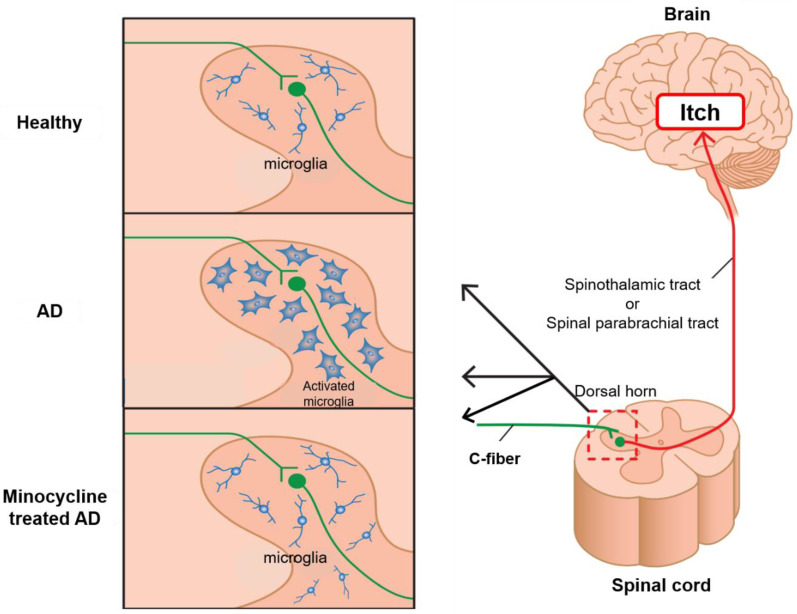
Antipruritic mechanism of minocycline in AD. The number of microglia is increased in the dorsal horn of the spinal cord of mice with AD. The administration of minocycline decreases the number of microglia in the spinal dorsal horn and suppresses itch and dermatitis.

Itch signals generated in the peripheral skin excite primary sensory nerve fibers, which relay signals to the brain via spinal dorsal horn neurons [47]. We previously demonstrated that the number of microglia, a type of glial cell, was increased in the spinal dorsal horn of AD mice. Furthermore, the intrathecal administration of minocycline (minomycin; Pfizer, New York, NY, USA), an inhibitor of microglial activation, to AD mice suppressed itch and attenuated dermatitis [48]. These findings suggest that microglia in the dorsal horn of the spinal cord are involved in intractable pruritus and may be one of the therapeutic targets for intractable pruritus (Figure 3). However, these antibiotics are off-label for itch.

### 2.5. Crotamiton

Crotamiton is a drug that is applied externally as an antipruritic, as well as an antiscabies measure. Crotamiton (Wako Pure Chemical Industries, Ltd., Osaka, Japan) was shown to be effective against histamine-, serotonin-, SLIGRL-NH_2_ (Protease activated receptor (PAR) 2 agonist)-, and chloroquine-induced itch and DRG responses [49,50]. In addition, it inhibited the responses of DRG neurons to histamine, chloroquine, and GSK1016790A, a TRPV4-selective agonist [50,51]. Therefore, crotamiton suppresses PAR2, TRPV1, TRP ankyrin (A) 1, and TRPV4-mediated itch [52].

### 2.6. PDE_4_ Inhibitor

PDE_4_, which catalyzes the conversion of cyclic adenosine 3′,5′-monophosphate (cAMP) to 5′-AMP, plays a critical role in the pathogenesis of inflammatory disorders [53]. OPA-15406 ointment (Difamilast, Otsuka Pharmaceutical Co., Ltd., Tokyo, Japan), a PDE_4_ inhibitor, ameliorated AD symptoms, including itch, in pediatric and adult AD patients [54,55,56]. Apremilast (Brand name Otezla (Amgen, CA, USA) among others) attenuated the symptoms of AD, hidradenitis suppurativa, and psoriasis, including itch [57,58,59,60,61,62,63]. In addition, itch and other symptoms in AD were mitigated by crisaborole ointment (Eucrisa, Pfizer, New York, NY, USA) [64,65,66,67,68]. A previous study demonstrated that crisaborole attenuated AD symptoms by modulating epidermal hyperplasia/proliferation and Th2 and Th17/Th22 transcriptional profiles in AD lesions [66]. E6005 (Roivant Sciences Ltd., Basel, Switzerland) also suppressed symptoms, including itch, in AD model mice and AD patients, as well as SLIGRL-NH_2_-induced symptoms [53,69,70]. The expression of cAMP was up-regulated in skin and DRG treated with E6005. Furthermore, capsaicin-induced DRG neuron responses were inhibited by E6005, as well as the cAMP elevator, forskolin. In addition, SLIGRL-NH_2_-induced DRG neuron responses were suppressed by E6005 and 8-bromo-cAMP, an analog of cAMP. Based on these findings, E6005 may increase cAMP levels and suppress itch via PAR2 [53,71].

There are various types of PDE_4_ inhibitors, and further studies are warranted to examine their mechanisms of action and potential applications to the treatment of diseases. OPA-15406 has been shown to improve AD symptoms, and its use is covered by health insurance [72], which is applicable for psoriasis vulgaris and psoriatic arthritis.

### 2.7. Botulinum Toxin A

Acetylcholine has been demonstrated to mediate pruritus in AD. Botulinum toxin type A (BT-A) blocks the release of acetylcholine and many other neurotransmitters from the presynaptic vesicle by deactivating soluble N-ethylmaleimide sensitive factor attachment protein receptor (SNARE) proteins. BT-A (trade name Botox (KC Pharmaceuticals, Pomona, CF, USA)) relieved itching in patients with conditions, such as lichen simplex chronicus (LSC), brachioradial pruritus, inverse psoriasis, post-burning itch, lichen planus, hypertrophic lichen planus, and postherpetic neuralgia [73,74,75]. In addition, there were statistically significant post-treatment reductions in both the severity strata for eczema area and severity index (EASI) score in LSC cases and the psoriasis area and severity index (PASI) score in inverse psoriasis cases [75]. In patients with Fox–Fordyce disease, treatment with BT-A significantly improved not only pruritus but also hyperhidrosis [76]. BT-A inhibited degranulation of mast cells and suppressed histaminergic itch [77,78]. Moreover, BT-A also inhibited mast cell independent chloroquine-induced itch [78]. Triamcinolone acetonide (TAC) is widely used to treat pathologic scars, but scar itch was significantly reduced by TAC combined with BT-A than TAC alone. In rat, postburn itch was mitigated by treatment with TAC, BT-A, and TAC/BT-A. TAC/BT-A treatment significantly decreased the density of IENFs in burn lesions relative to untreated burn lesions and downregulated the expressions of nerve growth factor (NGF) and TRPV1 [79].

From the above, we can infer that BT-A is effective for the treatment of histaminergic and non-histaminergic itch.

### 2.8. Herbal Medicines

Herbal medicines have long been used as antipruritics. Byakko-ka-ninjin (Tochimoto Tenkaido, Osaka, Japan), licorice gel, Yokukansan (YKS; Tsumura & Co., Tokyo, Japan), Xiao-Feng-Sang (Sheng Chang Pharmaceutical Company, Taipei, Taiwan), Pei Tu Qing Xin (PTQX; Jiangyin Tian Jiang Pharmaceutical Co., Ltd., Jiangyin, China), and Huanglian Jiedu decoction (HLJDT) are known to be effective for itch of AD [80,81,82,83,84,85].

YKS was shown to inhibit the infiltration of immune cells, such as mast cells and eosinophils, and attenuate itch in AD model mice. Moreover, serum corticosterone levels were decreased in AD model mice [82]. PTQX inhibited mast cell infiltration into the skin region in AD model mice, suppressing dermatitis and itch and reducing serum IgE levels. In addition, CD4^+^IL-4^+^ T cell differentiation and regulatory T cell (Treg) infiltration were found to be inhibited, which suggested that PTQX regulates T cell differentiation and the Th17/Treg balance [84]. AD model mice treated with HLJDT showed it ameliorated dermatitis, reduced cytokine production, and decreased expression of molecules required by dendritic cells (DC) to activate T cells, such as co-stimulatory molecules. Collectively, these findings indicate that HLJDT suppresses inflammation and itch by regulating the functions of DC [85].

The leaf extract of Rhamnus davurica (LERD), which is used to treat ich, has been reported to inhibit the Fyn/Syk pathway in mast cells, suggesting that it suppresses allergic activity, including itch [86]. Spirodelae Herba (SH; Medicinal Materials Company, Ulsan, Korea), which is an effective treatment for itch in AD, ameliorated AD symptoms by modulating the activation of the calcium ion channels Orai1 and TPRV4 and inhibiting mast cell degranulation [87].

In non-AD itch, Osthole (Sigma-Aldrich Corp., St. Louis, MO, USA), an active coumarin isolated from *Cnidium monnieri* (L.) Cusson, suppressed histamine-induced itch by inhibiting TRPV1 activation [88].

*Cnidium monnieri* (Cnidii Fructus; Tochimoto Tenkaido Co., Ltd., Osaka, Japan) and Unsei-in (Kanebo, Tokyo, Japan) are also effective against SP-induced itch. Nitric oxide (NO) enhanced SP-induced itch and Unsei-in reduced SP-induced itch by suppressing the expression of NO synthase in lesional skin [89,90].

Therefore, herbal medicines are effective treatments for not only AD, but also various types of itch.

## 3. Other Therapeutic Options for Itch

### 3.1. Phototherapy

Ultraviolet (UV)-based therapies, such as psoralen ultraviolet A (PUVA), narrowband-ultraviolet B (NB-UVB), and excimer lamps, are effective treatments for chronic pruritus in patients with AD and psoriasis [91,92,93]. In the guidelines for AD, phototherapy is indicated as a treatment for patients who do not respond to or cannot be controlled with steroid ointments, topical moisturizers, calcineurin inhibitors, and other treatments [94].

NB-UVB is a device with an irradiation range centered at 311 nm, which removes the harmful wavelengths present in UVB and is a highly effective treatment, while excimer lamps are local devices that generate rays at 308 nm. UVA (320–400 nm) is subdivided into UVA2 (320–340 nm) and UVA1 (340–400 nm); PUVA uses UVA1. UVA rays penetrate deeper than UVB, while UVB is more readily absorbed by DNA than UVA [95].

**Figure 4:**
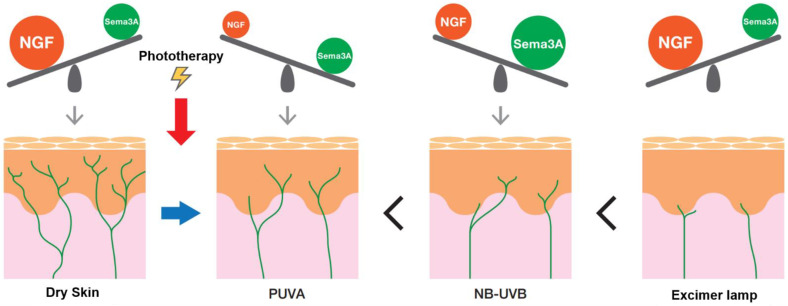
Antipruritic mechanism of phototherapy. In dry skin, the up-regulated expression of NGF and down-regulated expression of Sema3A increase the number of IENFs. When various types of phototherapy are used for dry skin, they inhibit IENF hyperplasia in the order of PUVA therapy < NB-UVB < excimer lamps. The inhibitory effects of PUVA and NB-UVB therapy on IENF proliferation are due to the down-regulated expression of nerve elongation factors and up-regulated expression of nerve repulsion factors in keratinocytes; however, excimer lamps act directly on IENFs and induce neurodegeneration without altering the expression of these axon guidance molecules.

We previously demonstrated that PUVA therapy exerted anti-pruritic effects by eliminating the hyperplasia of IENFs in AD patients and a dry skin model. Furthermore, the expression of nerve elongation factor (NGF) was down-regulated in the PUVA-treated group, whereas that of semaphorin 3A (Sema3A), a nerve repulsive factor, was significantly up-regulated in the skin of AD patients and slightly elevated in the skin of dry skin model mice [96,97]. In dry skin model mice, NB-UVB therapy exerted more potent quenching effects on IENFs than did PUVA, significantly down-regulated NGF expression, and significantly up-regulated Sema3A expression in the epidermis [97]. Excimer lamps suppressed itch more potently than did NB-UVB in AD model mice by suppressing IENFs, the number of which was increased in dry skin [97,98]. In contrast to NB-UVB and PUVA, excimer lamp therapy directly affected IENFs without altering the expression of axon guidance molecules and induced neurodegeneration, resulting in the disappearance of IENFs [98]. These findings suggest that PUVA and NB-UVB suppress itch by normalizing the balance of the expression of axon guidance molecules in epidermal keratinocytes, whereas excimer lamps act directly on nerve fibers to suppress itch (Figure 4).

Therefore, phototherapy is useful in the treatment of itch, while PUVA therapy requires the administration of soralen, as described above, and long-term PUVA therapy has been reported to increase the risk of skin carcinogenesis [99,100]. NB-UVB therapy does not significantly increase the risk of skin cancer but needs to be avoided in patients with photosensitivity [100].

### 3.2. Acupuncture Therapy

Acupuncture is used to treat pruritus. Symptoms, including itch, were attenuated by acupuncture in AD model animals and patients [101,102,103,104,105,106,107] and in uremic pruritus patients [108,109].

The antipruritic mechanism of acupuncture treatment at LI11 for AD was previously reported to be mediated by dynorphin in the spinal cord and the blockade of 5-HT_2_ receptors (R) and 5-HT_7_R to inhibit serotonin-induced itch [101,107]. Acupuncture treatment at LI11, PC6, HT7, SP10, ST36, and ear was also shown to be effective for histamine-induced itch, both therapeutically and prophylactically [110,111,112]. Brain-focused studies demonstrated that a decreased response in the putamen, an itch-evoked activation region, and part of the midcingulate cortex (pMCC) were involved in the suppressive effects of acupuncture at LI11, PC6, HT7, SP10, ST36, and HT3 on itch [106,112].

Itch in a dry skin model mouse induced by an acetone/diethyl ether and water treatment was also suppressed by acupuncture at LI4 and LI11. In this model, itch was inhibited by suppressing gastrin-releasing peptide receptor (GRPR) expression via the activation of KOR in the dorsal horn of the spinal cord [113].

A previous study reported that morphine-induced itch was ameliorated by acupuncture at LI11 and SP10 through the Toll-like receptor (TLR) 2/4-myeloid differentiation factor (MyD) 88-NF-κB pathway of the spinal cord [114]. Moreover, itch induced by one of the secondary bile acids, deoxycholic acid, was attenuated by acupuncture at LI4 and LI11 via suppressing the activation of microglia [115]. Thus, acupuncture therapy is useful for the treatment of itch caused by various factors.

## 4. Discussion

Itch (pruritus) is a self-defense system against external foreign objects and an alarm reaction that alerts the whole body, including the skin, of abnormalities. Histamine is one of the major itch mediators, but recent studies have showed histamine-dependent and histamine-independent pathways in transmitting itch. Other systems, including proteases, neuropeptides, cytokines, and opioids, and their cognate receptors, such as thermoreceptors, PARs, Mas-related G protein-coupled receptors (Mrgprs) and opioid receptors, are involved in the histamine-independent itch pathway. The sensation of itch is generated by the binding of itch-inducing substances to specific receptors on peripheral sensory afferent, such as unmyelinated C-fiber afferents and the myelinated Aδ fiber afferents. This excitatory information from sensory nerve fibers transmits itch signals by various neurotransmitters to secondary neurons distributed in the dorsal horn of the spinal cord. The information from the spinal cord passes through the thalamus and posterior parietal cortex and is ultimately processed at multiple regions of the brain [47,116,117]. Many kinds of mediators, receptors, and channels are involved in itch signaling among the skin nervous system, skin cells, and central nervous systems, including Mrgprs, TLR, cytokines, and TRP channels [47]. Therefore, we need to provide treatment tailored to the pathology, and treatment guidelines for troublesome itch have not yet been established.

Of the itch treatments described in this review, none are covered by insurance except for CsA, OPA-15406, and phototherapy. However, the insurance coverage described in this review has limitations from a global perspective. Moreover, NTP is currently available only in Japan and China. In addition, emollients also have a certain therapeutic effect, but patients often find the application of ointments cumbersome, which may reduce compliance [118]. In light of the above, even with the treatment methods presented here, topical medication should take into consideration the patient’s compliance with local preparations. Therefore, it is also important to provide guidance for patients on treatment methods.

## 5. Conclusions

This review summarized the antipruritic effects and mechanisms of action of treatment options for itch, such as antidepressants, antineuralgics, cyclosporine, antibiotics, crotamiton, PDE_4_ inhibitors, botulinum toxin type A, herbal medicine, phototherapy, and acupuncture. Itch is an equally or even more distressing sensation than pain and significantly reduces the QOL of patients. Recent advancements have been achieved in the establishment of objective assessments of itch and development of animal models, and the molecular and cellular mechanisms underlying itch are being elucidated. In the future, the antipruritic mechanisms of the treatments and novel agents introduced here will be clarified through the development of bidirectional clinical and basic medical research.

## Figures and Tables

**Table 1:** Therapeutic methods for troublesome itch.

Category	Therapeutic Methods	Mechanisms of Action
Oral therapy	Antidepressants • SSRIs (Paroxetine, Fluvoxamine, and *Sertraline*), NaSSA (*Mirtazapine*)	Inhibits reuptake of serotonin. Activates the descending inhibitory system in CNS.
	Analgesics • *Pregabalin*, Mirogabalin, Neurotropin	*Pregabalin* may inhibit calcium influx and the release of neurotransmitters, such as glutamate. Mirogabalin may inhibit calcium influx in the peripheral nervous system and suppress the release of neurotransmitter. Neurotropin inhibits neuropeptide release and decreases IENF density.
	Calcineurin inhibitor • Cyclosporine A	Activates T cells and inhibits immune cell infiltration. Inhibits the release of inflammatory cytokines. Decreases IENF density. Suppresses itch mediator receptor expression.
	Antibiotics • Roxithromycin, Clarithromycin, Minomycin	Anti-inflammatory effects. Immunomodulation. Minomycin suppresses microglia.
	PDE_4_ inhibitor • Apremilast	Unknown
	Herbal remedies • Yokukansan (YKS), Xiao-Feng-Sang, Pei Tu Qing Xin (PTQX), and Huanglian Jiedu decoction (HLJDT), leaf extract of Rhamnus davurica (LERD), Spirodelae Herba (SH), Osthole, *Cnidium monnieri*, and Unsei-in	YKS and PTQX inhibit the infiltration of immune cells. HLJDT regulates DC functions. LERD inhibits the Fyn/Syk pathway in mast cells. SH modulates the activation of Orai1 and TPRV4 and inhibits mast cell degranulation. Osthole inhibits TRPV1 activation. Unsei-in inhibits the expression of NO synthase.
Topical medication	Antidepressants • SNRI (Milnacipran)	Inhibits reuptake of serotonin. Activates the descending inhibitory system in CNS.
	Antibiotics • Josamycin	Inhibits *Staphylococcus aureus* colonization. Inhibits Th2 cell differentiation. ⇒suppresses IL-31 production.
	Crotamiton	Inhibits TRPV1, TRPA1, and TRPV4 on nerves.
	PDE_4_ inhibitors • OPA-15406, crisaborole, and E6005	Crisaborole modulates epidermal hyperplasia/proliferation and Th2 and Th17/Th22 transcriptional profiles. E6005 increases cAMP.
	Botulinum toxin type A	Blocks the release of acetylcholine and neurotransmitters from the presynaptic vesicle by deactivating SNARE proteins. Inhibits degranulation of mast cells. Normalizes the expression of nerve elongation factors and nerve repulsion factors. ⇒decreases IENF density.
Phototherapy	PUVA and NB-UVB	Normalizes the expression of nerve elongation factors and nerve repulsion factors. ⇒decreases IENF density.
	Excimer lamp	Decreases IENF density.
Acupuncture therapy		Treatment LI11 blocks 5-HT_2_R and 5-HT_7_R. Treatments LI11, PC6, HT7, SP10, ST36 and HT3 decrease responses in the putamen, pMCC. Treatments LI4 and LI11 suppress GRPR expression via KOR activation. Treatments LI11 and SP10 inhibit the TLR2/4-MyD88-NF-κB pathway. Treatments LI4 and LI11 suppress activation of microglia.

SSRIs = selective serotonin reuptake inhibitors, NaSSA = noradrenergic and specific serotonergic antidepressant, IENF = intraepidermal nerve fiber, TPRV = transient receptor potential vanilloid, NO = Nitric oxide, SNRI = serotonin noradrenaline reuptake inhibitors, TRPA = TRP ankyrin, cAMP = cyclic adenosine 3′,5′-monophosphate, SNARE = soluble N-ethylmaleimide sensitive factor attachment protein receptor, PUVA = psoralen ultraviolet A, NB-UVB = narrowband-ultraviolet B, 5-HT = 5-hydroxytryptamine, pMCC = part of the midcingulate cortex, GRPR = gastrin-releasing peptide receptor, TLR = Toll-like receptor, MyD88 = myeloid differentiation factor 88, NF-κB = nuclear factor-κ B.

## Data Availability

Data sharing not applicable.
